# Checklist of the families Scathophagidae, Fanniidae and Muscidae of Finland (Insecta, Diptera)

**DOI:** 10.3897/zookeys.441.7142

**Published:** 2014-09-19

**Authors:** Jere Kahanpää, Antti Haarto

**Affiliations:** 1 Finnish Museum of Natural History, Zoology Unit, P.O. Box 17, FI–00014 University of Helsinki, Finland; 2 Zoological Museum, Section of Biodiversity and Environmental Science, Department of Biology, University of Turku, FI–20014 Turku, Finland

**Keywords:** Species list, Finland, Diptera, biodiversity, faunistics

## Abstract

A revised checklist of the Scathophagidae, Fanniidae and Muscidae recorded from Finland is presented. *Phaonia amicula* Villeneuve, 1922 is noted from Finland for the first time.

## Introduction

Four families make up the traditional superfamily Muscoidea: Scathophagidae, Anthomyiidae, Fanniidae and Muscidae. The monophyly of the superfamily has been strongly questioned (Nirmala et al. 2001, Kutty et al. 2010) on the basis of DNA sequence analyses.

Three of the four families of Muscoidea are treated in this paper, the fourth (Anthomyiidae) is covered in a separate paper in this issue of ZooKeys. All three are important components of the Finnish fly fauna as can be seen from the large number of species involved and the fact that the species recorded from the country represent up to one fifth of the world fauna in the case of Scathophagidae.

Unfortunately the muscoid flies have been poorly collected and studied in Finland in comparison with many other Diptera families. Lauri Tiensuu concentrated on these families during the 1930s (1935, 1938, 1939) and Walter Hackman (1956) reviewed the Finnish Scathophagidae. During the last ten years, both the fanniids (Kahanpää and Haarto 2013) and the muscids (Kahanpää 2013) have attracted some attention in the country. The number of species known from the country has increased markedly since the year 2000.

The Finnish muscoid species were last listed by Hackman (1980).

**Table 1.** Number of species by family.

Family	Number of species in			Level of knowledge
	World (Pape et al. 2011)	Europe	Finland	
Scathophagidae	419	158	85–86	good
Fanniidae	359	83	61	average
Muscidae	5218	572	307–309	average

## Checklist

suborder Brachycera Macquart, 1834

clade Eremoneura Lameere, 1906

clade Cyclorrhapha Brauer, 1863

infraorder Schizophora Becher, 1882

clade Muscaria Enderlein, 1936

parvorder Calyptratae Robineau-Desvoidy, 1830

superfamily Muscoidea Latreille, 1802

**SCATHOPHAGIDAE** Robineau-Desvoidy, 1830

DELININAE Séguy, 1952

***DELINA*** Robineau-Desvoidy, 1830

*Delina nigrita* (Fallén, 1819)

***LEPTOPA*** Zetterstedt, 1838

*Leptopa filiformis* Zetterstedt, 1838

***MICROPSELAPHA*** Becker, 1894

*Micropselapha filiformis* (Zetterstedt, 1846)

***PARALLELOMMA*** Strobl, 1894

= *Chylizosoma* Hendel, 1924

*Parallelomma medium* Becker, 1894

? *Parallelomma paridis* Hering, 1923 (see Notes)

*Parallelomma sellatum* (Hackman, 1956)

*Parallelomma vittatum* (Meigen, 1826)

***PHROSIA*** Robineau-Desvoidy, 1830

*Phrosia albilabris* (Fabricius, 1805)

SCATHOPHAGINAE Robineau-Desvoidy, 1830

***ACANTHOCNEMA*** Becker, 1894

**sg. *Clinoceroides*** Hendel, 1917

*Acanthocnema glaucescens* (Loew, 1864)

= *nigripes* Ringdahl, 1936

***ACEROCNEMA*** Becker, 1894

*Acerocnema macrocera* (Meigen, 1826)

= *tiefi* Becker, 1894

= *pokornyi* Becker, 1894

***ALLOMYELLA*** Malloch, 1923

*Allomyella albipennis* (Zetterstedt, 1838)

*Allomyella frigida* (Holmgren, 1883)

= *portenkoi* (Stackelberg, 1952) misid.

***BOSTRICHOPYGA*** Becker, 1894

*Bostrichopyga crassipes* (Zetterstedt, 1838)

***CHAETOSA*** Coquillett, 1898

*Chaetosa punctipes* (Meigen, 1826)

***CLEIGASTRA*** Macquart, 1835

= *Cnemopogon* Rondani, 1856

*Cleigastra apicalis* (Meigen, 1826)

***CONISTERNUM*** Strobl, 1894

= *Coniosternum* Becker, 1894

*Conisternum lapponicum* (Ringdahl, 1920)

*Conisternum obscurum* (Fallén, 1819)

*Conisternum tinctinerve* (Becker, 1894)

***CORDILURA*** Fallén, 1810

= *Cordylura* Meigen, 1826 emend.

**sg. *Cordilura*** Fallén, 1810

*Cordilura aberrans* Becker, 1894

*Cordilura aemula* Collin, 1958

*Cordilura atrata* Zetterstedt, 1846

*Cordilura ciliata* Meigen, 1826

*Cordilura picipes* Meigen, 1826

*Cordilura picticornis* Loew, 1864

*Cordilura proboscidea* Zetterstedt, 1838

*Cordilura pubera* (Linnaeus, 1758)

*Cordilura pudica* Meigen, 1826

*Cordilura rufimana* Meigen, 1826

*Cordilura socialis* (Becker, 1894)

= *freyi* Hackman, 1956

**sg. *Cordilurina*** James, 1955

= *Parallelomma* Becker, 1894 preocc.

*Cordilura albipes* Fallén, 1819

*Cordilura fuscipes* Zetterstedt, 1838

**sg. *Scoliaphleps*** Becker, 1894

*Cordilura ustulata* Zetterstedt, 1838

= *melanacra* Loew, 1873

***COSMETOPUS*** Becker, 1894

*Cosmetopus dentimana* (Zetterstedt, 1838)

= *fulvipes* (Zetterstedt, 1838)

*Cosmetopus longa* (Walker, 1849)

= *bergrothi* Becker, 1900 in part

= *fulvipes* auct. nec (Zetterstedt, 1838)

*Cosmetopus ringdahli* Andersson, 1974

= *bergrothi* Becker, 1900 in part

***ERNONEURA*** Becker, 1894

*Ernoneura argus* (Zetterstedt, 1838)

***GONARCTICUS*** Becker, 1894

*Gonarcticus abdominalis* (Zetterstedt, 1846)

***GONATHERUS*** Rondani, 1856

*Gonatherus planiceps* (Fallén, 1826)

***GIMNOMERA*** Rondani, 1866

= *Cochliarium* Becker, 1894

*Gimnomera albipila* (Zetterstedt, 1846)

*Gimnomera cuneiventris* (Zetterstedt, 1846)

*Gimnomera dorsata* (Zetterstedt, 1838)

*Gimnomera hirta* Hendel, 1930

*Gimnomera tarsea* (Fallén, 1819)

***HEXAMITOCERA*** Becker, 1894

*Hexamitocera loxoceratum* (Fallén, 1826)

***HYDROMYZA*** Fallén, 1823

*Hydromyza livens* (Fabricius, 1794)

***MEGAPHTHALMA*** Becker, 1894

*Megaphthalma pallida* (Fallén, 1819)

***MEGAPHTHALMOIDES*** Ringdahl, 1936

*Megaphthalmoides unilineatus* (Zetterstedt, 1838)

***MICROPROSOPA*** Strobl, 1894

*Microprosopa haemorrhoidalis* (Meigen, 1826)

*Microprosopa lacteipennis* Ringdahl, 1920

*Microprosopa lineata* (Zetterstedt, 1838)

*Microprosopa pallidicauda* (Zetterstedt, 1838)

***NANNA*** Strobl, 1894

= *Amaurosoma* Becker, 1894

*Nanna armillata* (Zetterstedt, 1846)

*Nanna articulata* (Becker, 1894)

*Nanna bispinosa* (Malloch, 1920)

*Nanna brevifrons* (Zetterstedt, 1838)

*Nanna fasciata* (Meigen, 1826)

*Nanna flavipes* (Fallén, 1819)

= *minuta* (Becker, 1894)

?= *multisetosa* (Hackman, 1956)

*Nanna inermis* (Becker, 1894)

*Nanna leucostoma* (Zetterstedt, 1846)

*Nanna tibiella* (Zetterstedt, 1838)

= *nigripes* (Zetterstedt, 1846)

***NORELLIA*** Robineau-Desvoidy, 1830

*Norellia tipularia* (Fabricius, 1794) (see Notes)

***NORELLISOMA*** Hendel, 1910

*Norellisoma lituratum* (Wiedemann, 1826)

*Norellisoma spinimanum* (Fallén, 1819)

***OKENIELLA*** Hendel, 1907

*Okeniella caudata* (Zetterstedt, 1838)

*Okeniella dasyprocta* (Loew, 1864)

***ORTHACHETA*** Becker, 1894

*Orthacheta pilosa* (Zetterstedt, 1838)

***PLEUROCHAETELLA*** Vockeroth, 1965

*Pleurochaetella simplicipes* (Becker, 1900)

***POGONOTA*** Zetterstedt, 1860

**sg. *Pogonota*** Zetterstedt, 1860

*Pogonota barbata* (Zetterstedt, 1838)

**sg. *Lasioscelus*** Becker, 1894

*Pogonota immunda* (Zetterstedt, 1838)

= *clavatus* (Zetterstedt, 1846)

*Pogonota sahlbergi* Becker, 1900

***SCATHOPHAGA*** Meigen, 1803

= *Scatophaga* Frabricius, 1805 emend.

= *Scopeuma* Meigen, 1800 suppr.

= *Scatomyza* Fallén, 1810

*Scathophaga apicalis* Curtis *in* Ross, 1835

*Scathophaga furcata* (Say, 1823)

*Scathophaga incola* Becker, 1900

*Scathophaga inquinata* (Meigen, 1826)

= *analis* Meigen, 1826

*Scathophaga litorea* (Fallén, 1819)

*Scathophaga lutaria* (Fabricius, 1794)

*Scathophaga obscurinervis* Becker, 1900

*Scathophaga pictipennis* Oldenberg, 1923

*Scathophaga scybalaria* (Linnaeus, 1758)

*Scathophaga stercoraria* (Linnaeus, 1758)

*Scathophaga suilla* (Fabricius, 1794)

***SPAZIPHORA*** Rondani, 1856

*Spaziphora hydromyzina* (Fallén, 1819)

= *fascipes* (Becker, 1894)

***STAEGERIA*** Rondani, 1856

*Staegeria kunzei* (Zetterstedt, 1821)

***TRICHOPALPUS*** Rondani, 1856

*Trichopalpus fraternus* (Meigen, 1826)

*Trichopalpus nigribasis* Curran, 1927

= *pilirostris* (Ringdahl, 1936)

*Trichopalpus obscurellus* (Zetterstedt, 1846)

= *subarcticus* (Ringdahl, 1936)

**FANNIIDAE** Schnabl & Dziezicki, 1911

***FANNIA*** Robineau-Desvoidy, 1830

= *Homalomyia* Bouché, 1834

= *Coelomyia* Haliday, 1840

*Fannia aethiops* Malloch, 1913

*Fannia alpina* Pont, 1970

*Fannia armata* (Meigen, 1826)

*Fannia atra* (Stein, 1895)

*Fannia canicularis* (Linnaeus, 1761)

*Fannia carbonaria* (Meigen, 1826)

*Fannia carbonella* (Stein, 1895)

*Fannia coracina* (Loew, 1873)

*Fannia corvina* (Verrall, 1892)

= *halterata* Ringdahl, 1918

*Fannia cothurnata* (Loew, 1873)

*Fannia difficilis* (Stein, 1895)

*Fannia fuscitibia* Stein, 1920

*Fannia fuscula* (Fallén, 1825)

*Fannia genualis* (Stein, 1895)

*Fannia gotlandica* Ringdahl, 1926

*Fannia hirticeps* (Stein, 1892)

*Fannia hirundinis* Ringdahl, 1948

*Fannia immutica* Collin, 1939

*Fannia incisurata* (Zetterstedt, 1838)

*Fannia latifrontalis* Hennig, 1955

*Fannia latipalpis* (Stein, 1892)

*Fannia lepida* (Wiedemann, 1817)

= *mutica* (Zetterstedt, 1845)

*Fannia leucosticta* (Meigen, 1838)

*Fannia limbata* (Tiensuu, 1938)

= *berolinensis* Hennig, 1955

*Fannia lucidula* (Zetterstedt, 1860)

= *glaucescens* auct. nec (Zetterstedt, 1845)

*Fannia lugubrina* (Zetterstedt, 1838)

*Fannia lustrator* (Harris, 1780)

= *hamata* (Macquart, 1835)

*Fannia manicata* (Meigen, 1826)

*Fannia melania* (Dufour, 1839)

= *ciliata* (Stein, 1895)

*Fannia metallipennis* (Zetterstedt, 1838)

= *kowarzi* (Verrall, 1892)

*Fannia minutipalpis* (Stein, 1895)

*Fannia mollissima* (Haliday, 1840)

= *spathulata* (Zetterstedt, 1845)

*Fannia monilis* (Haliday, 1838)

*Fannia pallitibia* (Rondani, 1866)

*Fannia parva* (Stein, 1895)

*Fannia pauli* Pont, 1997

= *nitida* (Stein, 1895) preocc.

*Fannia polychaeta* (Stein, 1895)

*Fannia postica* (Stein, 1895)

*Fannia posticata* (Meigen, 1826)

= *pretiosa* (Schiner, 1862)

*Fannia rabdionata* Karl, 1940

*Fannia ringdahlana* Collin, 1939

= *umbrosa* auct. nec (Stein, 1895)

*Fannia rondanii* (Strobl, 1893)

= *carbonaria* (Rondani, 1871) preocc.

= *aerea* misid.

*Fannia scalaris* (Fabricius, 1794)

= *subscalaris* Zimin, 1946

*Fannia serena* (Fallén, 1825)

*Fannia similis* (Stein, 1895)

*Fannia slovaca* Gregor & Rozkošný, 2005

*Fannia sociella* (Zetterstedt, 1845)

*Fannia spathiophora* Malloch 1918

= *nodulosa* Ringdahl, 1926

*Fannia speciosa* (Villeneuve, 1898)

*Fannia stigi* Rognes, 1982

*Fannia subatripes* d’Assis-Fonseca, 1967

*Fannia subpellucens* (Zetterstedt, 1845)

*Fannia subpubescens* Collin, 1958

*Fannia tuberculata* (Zetterstedt, 1849)

*Fannia umbratica* Collin, 1939

*Fannia umbrosa* (Stein, 1895)

= *subumbrosa* Ringdahl, 1934

*Fannia verrallii* (Stein, 1895)

*Fannia vesparia* (Meade, 1891)

*Fannia vespertilionis* Ringdahl, 1934

***PIEZURA*** Rondani, 1844

= *Platycoenosia* Strobl, 1894

*Piezura graminicola* (Zetterstedt, 1846)

= *boletorum* (Rondani, 1866)

= *mikii* (Strobl, 1894)

*Piezura pardalina* (Rondani, 1866)

= *graminicola* auct. nec (Zetterstedt, 1846)

**MUSCIDAE** Latreille, 1802

ACHANTHIPTERINAE Hennig, 1962

***ACHANTHIPTERA*** Rondani, 1856

*Achanthiptera rohrelliformis* (Robineau-Desvoidy, 1830)

= *inanis* (Fallén, 1825) preocc.

COENOSIINAE Verrall, 1888

tribe Coenosiini Verrall, 1888

***COENOSIA*** Meigen, 1826

= *Caricea* Robineau-Desvoidy, 1830

= *Oplogaster* Rondani, 1856

= *Dexiopsis* Pokorny, 1893

*Coenosia acuminata* Strobl, 1898

= *annulipes* Ringdahl, 1932

*Coenosia agromyzina* (Fallén, 1825)

*Coenosia ambulans* Meigen, 1826

*Coenosia bilineella* (Zetterstedt, 1838)

*Coenosia campestris* (Meigen, 1830)

= *sexnotata* auct. nec Meigen, 1826

? *Coenosia comita* (Huckett, 1936) (see Notes)

= *ovulifera* Tiensuu, 1938

*Coenosia dealbata* (Zetterstedt, 1838)

= *fulvicornis* (Zetterstedt, 1845)

*Coenosia flavimana* (Zetterstedt, 1845)

= *albatella* (Zetterstedt, 1849)

*Coenosia humilis* Meigen, 1826

*Coenosia intermedia* (Fallén, 1825)

*Coenosia lacteipennis* (Zetterstedt, 1845)

*Coenosia lineatipes* (Zetterstedt, 1845)

= *albicornis* misid.

*Coenosia means* Meigen, 1826

*Coenosia mollicula* (Fallén, 1825)

*Coenosia octopunctata* (Zetterstedt, 1838)

*Coenosia paludis* Tiensuu, 1939

*Coenosia pedella* (Fallén, 1825)

= *decipiens* Meigen, 1826

*Coenosia perpusilla* Meigen, 1826

*Coenosia pudorosa* Collin, 1953

*Coenosia pulicaria* (Zetterstedt, 1845)

*Coenosia pumila* (Fallén, 1825)

*Coenosia pygmaea* (Zetterstedt, 1845)

*Coenosia ruficornis* Macquart, 1835

= *litoralis* (Zetterstedt, 1846)

*Coenosia rufipalpis* Meigen, 1826

= *flavicauda* Ringdahl, 1937

*Coenosia sallae* Tiensuu, 1938

*Coenosia testacea* (Robineau-Desvoidy, 1830)

= *tricolor* (Zetterstedt, 1845)

= *alleni* d’Assis-Fonseca, 1966

*Coenosia tigrina* (Fabricius, 1775)

*Coenosia trilineella* (Zetterstedt, 1838)

= *trilineata* emend.

*Coenosia verralli* Collin, 1953

= *steini* Verrall, 1912 preocc.

***LIMNOSPILA*** Schnabl, 1902

*Limnospila albifrons* (Zetterstedt, 1849)

***LISPOCEPHALA*** Pokorny, 1893

*Lispocephala alma* (Meigen, 1826)

*Lispocephala erythrocera* (Robineau-Desvoidy, 1830)

*Lispocephala falculata* Collin, 1963

*Lispocephala fuscitibia* Ringdahl, 1944

*Lispocephala pallipalpis* (Zetterstedt, 1845)

*Lispocephala spuria* (Zetterstedt, 1838)

= *vitripennis* Ringdahl, 1951

*Lispocephala verna* (Fabricius, 1794)

***MACRORCHIS*** Rondani, 1877

*Macrorchis meditata* (Fallén, 1825)

***PSEUDOCOENOSIA*** Stein, 1916

*Pseudocoenosia abnormis* Stein, 1916

*Pseudocoenosia solitaria* (Zetterstedt, 1838)

= *longicauda* (Zetterstedt, 1860)

***SCHOENOMYZA*** Haliday, 1833

*Schoenomyza litorella* (Fallén, 1823)

tribe Limnophoriini Villeneuve, 1902

***LIMNOPHORA*** Robineau-Desvoidy, 1830

*Limnophora nigripes* (Robineau-Desvoidy, 1830)

*Limnophora pandellei* Séguy 1923 (see Notes)

*Limnophora riparia* (Fallén, 1824)

*Limnophora rotundata* Collin, 1930

*Limnophora tigrina* (Am Stein, 1860)

= *notata* (Fallén, 1823) preocc.

*Limnophora triangula* (Fallén, 1825)

*Limnophora uniseta* Stein, 1916

***LISPE*** Latreille, 1796

*Lispe consanguinea* Loew, 1858

*Lispe hydromyzina* Fallén, 1825

*Lispe litorea* Fallén, 1825

*Lispe melaleuca* Loew, 1847

*Lispe pygmaea* Fallén, 1825

*Lispe tentaculata* (De Geer, 1776)

*Lispe uliginosa* Fallén, 1825

***SPILOGONA*** Schnabl, 1911

*Spilogona aerea* (Fallén, 1825)

*Spilogona albisquama* (Ringdahl, 1932)

*Spilogona alpica* (Zetterstedt, 1845)

*Spilogona arenosa* (Ringdahl, 1918)

*Spilogona atrisquamula* Hennig, 1959

*Spilogona baltica* (Ringdahl, 1918)

*Spilogona brunneifrons* Ringdahl, 1931

*Spilogona brunneisquama* (Zetterstedt, 1845)

*Spilogona carbonella* (Zetterstedt, 1845)

*Spilogona contractifrons* (Zetterstedt, 1838)

*Spilogona denigrata* (Meigen, 1826)

*Spilogona depressiuscula* (Zetterstedt, 1838)

*Spilogona depressula* (Zetterstedt, 1845)

*Spilogona dispar* (Fallén, 1823)

= *funeralis* Rondani, 1866

*Spilogona falleni* Pont, 1984

= *litorea* auct. nec (Fallén, 1823)

*Spilogona krogerusi* (Ringdahl, 1941)

= *micans* misid.

*Spilogona leucogaster* (Zetterstedt, 1838)

*Spilogona malaisei* (Ringdahl, 1920)

*Spilogona marginifera* Hennig, 1959

= *marginalis* (Fallén, 1824) preocc.

*Spilogona meadei* (Schnabl, 1915)

*Spilogona megastoma* (Boheman, 1866)

*Spilogona nigriventris* (Zetterstedt, 1845)

*Spilogona nitidicauda* (Schnabl, 1911)

*Spilogona novemmaculata* (Zetterstedt, 1860)

*Spilogona opaca* (Schnabl, 1915)

*Spilogona pacifica* (Meigen, 1826)

= *vana* (Zetterstedt, 1845)

*Spilogona palmeni* (Ringdahl, 1935)

*Spilogona pseudodispar* (Frey, 1915)

= *spinitibia* (Ringdahl, 1918)

*Spilogona pusilla* (Huckett, 1932)

*Spilogona quinquelineata* (Zetterstedt, 1838)

*Spilogona semiglobosa* (Ringdahl, 1916)

*Spilogona sororcula* (Zetterstedt, 1845)

= *zetterstedti* (Ringdahl, 1918) preocc.

*Spilogona spectabilis* (Tiensuu, 1938)

*Spilogona surda* (Zetterstedt, 1845)

*Spilogona tenuis* Hennig, 1959

*Spilogona tornensis* (Ringdahl, 1926)

*Spilogona triangulifera* (Zetterstedt, 1838)

*Spilogona trianguligera* (Zetterstedt, 1838)

= *insularis* (Collin, 1921)

*Spilogona trigonata* (Zetterstedt, 1838)

*Spilogona tundrae* (Schnabl, 1915)

= *macropyga* (Frey, 1915)

*Spilogona tundrica* (Schnabl & Dziedzicki, 1911)

*Spilogona varsaviensis* (Schnabl & Dziedzicki, 1911)

= *glauca* (Stein, 1916)

*Spilogona veterrima* (Zetterstedt, 1845)

***VILLENEUVIA*** Schnabl & Dziedzicki, 1911

*Villeneuvia aestuum* (Villeneuve, 1902)

AZELIINAE Robineau-Desvoidy, 1830

tribe Azeliini Robineau-Desvoidy, 1830

***AZELIA*** Robineau-Desvoidy, 1830

*Azelia aterrima* (Meigen, 1826)

*Azelia cilipes* (Haliday, 1838)

*Azelia gibbera* (Meigen, 1826)

*Azelia monodactyla* Loew, 1874

*Azelia nebulosa* Robineau-Desvoidy, 1830

= *macquarti* (Staeger in Schiødte, 1843)

*Azelia trigonica* Hennig, 1956

= *nuda* Hennig, 1956

*Azelia triquetra* (Wiedemann, 1817)

*Azelia zetterstedtii* Rondani, 1866

***DRYMEIA*** Meigen, 1826

= *Pogonomyia* Rondani, 1871

= *Trichopticoides* Ringdahl, 1931

*Drymeia hamata* (Fallén, 1823)

*Drymeia tetra* (Meigen, 1826)

*Drymeia vicana* (Harris, 1780)

= *decolor* (Fallén, 1824)

***HYDROTAEA*** Robineau-Desvoidy, 1830

= *Ophyra* Robineau-Desvoidy, 1830

= *Lasiops* Meigen, 1838

*Hydrotaea aenescens* (Wiedemann, 1830)

*Hydrotaea albipuncta* (Zetterstedt, 1845)

*Hydrotaea anxia* (Zetterstedt, 1838)

= *bispinosa* (Zetterstedt, 1845)

*Hydrotaea armipes* (Fallén, 1825)

= *occulta* (Meigen, 1826)

*Hydrotaea basdeni* Collin, 1939

*Hydrotaea borussica* Stein, 1899

*Hydrotaea cyrtoneurina* (Zetterstedt, 1845)

*Hydrotaea dentipes* (Fabricius, 1805)

*Hydrotaea diabolus* (Harrris, 1780)

= *ciliata* (Fabricius, 1794) preocc.

= *bimaculata* (Meigen, 1826)

*Hydrotaea floccosa* Macquart, 1835

= *armipes* auct. nec (Fallén, 1825)

*Hydrotaea ignava* (Harrris, 1780)

= *leucostoma* (Wiedemann, 1817)

*Hydrotaea irritans* (Fallén, 1823)

*Hydrotaea meridionalis* Portschinsky, 1882

*Hydrotaea meteorica* (Linnaeus, 1758)

*Hydrotaea militaris* (Meigen, 1826)

*Hydrotaea nidicola* Malloch, 1925

*Hydrotaea palaestrica* (Meigen, 1826)

*Hydrotaea pandellei* Stein, 1899

*Hydrotaea parva* Meade, 1889

*Hydrotaea pellucens* Portschinsky, 1879

*Hydrotaea pilipes* Stein, 1903

*Hydrotaea pilitibia* Stein, 1916

*Hydrotaea ringdahli* Stein, 1916

*Hydrotaea scambus* (Zetterstedt, 1838)

*Hydrotaea similis* Meade, 1887

*Hydrotaea tuberculata* Rondani, 1866

*Hydrotaea velutina* Robineau-Desvoidy, 1830

***POTAMIA*** Robineau-Desvoidy, 1830

= *Dendrophaonia* Malloch, 1923

*Potamia littoralis* Robineau-Desvoidy, 1830

= *querceti* (Bouché, 1834)

***THRICOPS*** Rondani, 1856

= *Alleostylus* Schnabl, 1888

*Thricops aculeipes* (Zetterstedt, 1838)

*Thricops albibasalis* (Zetterstedt, 1849)

= *sudeticus* auct. nec (Schnabl, 1888)

*Thricops coquilletti* (Malloch, 1920)

*Thricops cunctans* (Meigen, 1826)

= *hirsutulus* auct. nec (Zetterstedt, 1838)

*Thricops diaphanus* (Wiedemann, 1817)

*Thricops foveolatus* (Zetterstedt, 1845)

*Thricops genarum* (Zetterstedt, 1838)

= *sundewalli* (Zetterstedt, 1845)

*Thricops hirtulus* (Zetterstedt, 1838)

= *subrostratus* (Zetterstedt, 1845)

*Thricops innocuus* (Zetterstedt, 1838)

*Thricops lividiventris* (Zetterstedt, 1845)

*Thricops longipes* (Zetterstedt, 1845)

*Thricops nigrifrons* (Robineau-Desvoidy, 1830)

*Thricops nigritellus* (Zetterstedt, 1838)

*Thricops rostratus* (Meade, 1882)

*Thricops rufisquamus* (Schnabl, 1915)

= *penicillatus* (Ringdahl, 1926)

*Thricops semicinereus* (Wiedemann, 1817)

*Thricops separ* (Zetterstedt, 1845)

*Thricops simplex* (Wiedemann, 1817)

tribe Reinwardtiini Brauer & Bergenstamm, 1889

***MUSCINA*** Robineau-Desvoidy, 1830

*Muscina angustifrons* (Loew, 1858)

*Muscina levida* (Harris, 1780)

= *assimilis* (Fallén, 1823)

*Muscina pascuorum* (Meigen, 1826)

*Muscina prolapsa* (Harris, 1780)

= *pabulorum* (Fallén, 1817)

*Muscina stabulans* (Fallén, 1817)

MUSCINAE Latreille, 1802

tribe Muscini Latreille, 1802

***EUDASYPHORA*** Townsend, 1911

*Eudasyphora cyanicolor* (Zetterstedt, 1845)

*Eudasyphora zimini* (Hennig, 1963)

***MESEMBRINA*** Meigen, 1826

= *Hypodermodes* Townsend, 1912

*Mesembrina intermedia* Zetterstedt, 1848?

*Mesembrina meridiana* (Linnaeus, 1758)

*Mesembrina mystacea* (Linnaeus, 1758)

*Mesembrina resplendens* Wahlberg, 1844

***MORELLIA*** Robineau-Desvoidy, 1830

**sg. *Morellia*** Robineau-Desvoidy, 1830

*Morellia aenescens* Robineau-Desvoidy, 1830

*Morellia hortorum* (Fallén, 1817)

*Morellia podagrica* (Loew, 1857)

**sg. *Ziminiella*** Nihei & Carvalho, 2007

*Morellia simplex* (Loew, 1857)

***MUSCA*** Linnaeus, 1758

*Musca autumnalis* De Geer, 1776

= *corvina* Fabricius, 1781

*Musca domestica* Linnaeus, 1758

*Musca tempestiva* Fallén, 1817

***NEOMYIA*** Walker, 1859

= *Orthellia* Robineau-Desvoidy, 1863

*Neomyia cornicina* (Fabricius, 1781)

= *caesarion* (Meigen, 1826)

= *fennica* (Frey, 1909)

*Neomyia viridescens* (Robineau-Desvoidy, 1830)

= *cornicina* auct. nec (Fabricius, 1781)

***POLIETES*** Rondani, 1866

*Polietes domitor* (Harris, 1780)

= *albolineatus* (Fallén, 1823)

*Polietes lardarius* (Fabricius, 1781)

*Polietes nigrolimbatus* (von Bonsdorff, 1866)

*Polietes steinii* (Ringdahl, 1913)

***PYRELLIA*** Robineau-Desvoidy, 1830

*Pyrellia vivida* Robineau-Desvoidy, 1830

= *cadaverina* auct. nec (Linnaeus, 1758)

tribe Stomoxyini Meigen, 1824

***HAEMATOBIA*** Le Peletier & Serville, 1828

= *Lyperosia* Rondani, 1856

*Haematobia irritans* (Linnaeus, 1758)

***HAEMATOBOSCA*** Bezzi, 1907

*Haematobosca alcis* Snow, 1891

= *crassipalpis* (Ringdahl, 1920)

*Haematobosca stimulans* (Meigen, 1824)

***STOMOXYS*** Geoffroy, 1762

*Stomoxys calcitrans* (Linnaeus, 1758)

MYDAEINAE Verrall, 1888

***GRAPHOMYA*** Robineau-Desvoidy, 1830

*Graphomya maculata* (Scopoli, 1763)

*Graphomya minor* Robineau-Desvoidy, 1830

***GYMNODIA*** Robineau-Desvoidy, 1863

= *Brontaea* Kowarz, 1873

*Gymnodia humilis* (Zetterstedt, 1860)

***HEBECNEMA*** Schnabl, 1889

*Hebecnema fumosa* (Meigen, 1826)

*Hebecnema nigra* (Robineau-Desvoidy, 1830)

= *vespertina* auct. nec (Fallén, 1823)

*Hebecnema nigricolor* (Fallén, 1825)

*Hebecnema umbratica* (Meigen, 1826)

*Hebecnema vespertina* (Fallén, 1823)

= *affinis* Malloch, 1921

***MYDAEA*** Robineau-Desvoidy, 1830

*Mydaea affinis* Meade, 1891

= *discimana* Malloch, 1920

*Mydaea ancilla* (Meigen, 1826)

*Mydaea anicula* (Zetterstedt, 1860)

*Mydaea corni* Scopoli, 1763

= *pagana* (Fabricius, 1794) preocc.

= *scutellaris* Robineau-Desvoidy, 1830

*Mydaea deserta* (Zetterstedt, 1845)

*Mydaea detrita* (Zetterstedt, 1845)

= *electa* (Zetterstedt, 1860)

*Mydaea humeralis* Robineau-Desvoidy, 1830

= *tincta* (Zetterstedt, 1845)

*Mydaea nebulosa* (Stein, 1893)

*Mydaea obscurella* Malloch, 1921

= *bengtssoni* Ringdahl, 1924

*Mydaea orthonevra* (Macquart, 1835)

= *detrita* auct. nec (Zetterstedt, 1845)

*Mydaea palpalis* Stein, 1916

*Mydaea setifemur* Ringdahl, 1924

*Mydaea sootryeni* Ringdahl, 1928

*Mydaea urbana* (Meigen, 1826)

***MYOSPILA*** Rondani, 1856

*Myospila bimaculata* (Macquart, 1834)

*Myospila meditabunda* (Fabricius, 1781)

***OPSOLASIA*** Coquillett, 1910

*Opsolasia orichalcea* (Zetterstedt, 1849)

PHAONIINAE Malloch, 1917

tribe Phaoniini Malloch, 1917

***HELINA*** Robineau-Desvoidy, 1830

*Helina abdominalis* (Zetterstedt, 1846)

*Helina allotalla* (Meigen, 1830)

*Helina annosa* (Zetterstedt, 1838)

*Helina atricolor* (Fallén, 1825)

= *denudata* (Zetterstedt, 1845)

= *denutata* emend.

*Helina bohemani* (Ringdahl, 1916)

*Helina celsa* (Harris, 1780)

= *quadrimaculata* (Fallén, 1823) preocc.

= *quadrimaculella* Hennig, 1957

? *Helina ciliata* Karl, 1929 (see Notes)

*Helina ciliatocosta* (Zetterstedt, 1845)

= *ciliatocostata* emend.

*Helina cilipes* (Schnabl, 1902)

*Helina cinerella* (van der Wulp, 1867)

= *vanderwulpi* (Schnabl, 1888)

= *calceata* misid.

*Helina confinis* (Fallén, 1825)

= *anceps* (Zetterstedt, 1838)

*Helina consimilis* (Fallén, 1825)

*Helina cothurnata* (Rondani, 1866)

= *obscuripes* auct. nec (Zetterstedt, 1845)

*Helina daicles* (Walker, 1849)

= *binotata* (Zetterstedt, 1845)

*Helina depuncta* (Fallén, 1825)

*Helina evecta* (Harris, 1780)

= *lucorum* (Fallén, 1823) preocc.

= *laetifica* (Robineau-Desvoidy, 1830)

*Helina flavisquama* (Zetterstedt, 1849)

*Helina fratercula* (Zetterstedt, 1845)

*Helina fulvisquama* (Zetterstedt, 1845)

*Helina impuncta* (Fallén, 1825)

*Helina latitarsis* Ringdahl, 1924

*Helina laxifrons* (Zetterstedt, 1860)

*Helina longicornis* (Zetterstedt, 1838)

*Helina luteisquama* (Zetterstedt, 1845)

*Helina maculipennis* (Zetterstedt, 1845)

= *obscuripes* (Zetterstedt, 1845)

*Helina obscurata* (Meigen, 1826)

*Helina pertusa* (Meigen, 1826)

*Helina protuberans* (Zetterstedt, 1845)

*Helina pubiseta* (Zetterstedt, 1845)

*Helina quadrinotata* (Meigen, 1826)

*Helina quadrum* (Fabricius, 1805)

*Helina reversio* (Harris, 1780)

= *duplicata* (Meigen, 1826)

= *duplaris* auct. nec (Zetterstedt, 1845)

= *communis* (Robineau-Desvoidy, 1830)

*Helina setiventris* Ringdahl, 1924

*Helina sexmaculata* (Preyssler, 1791)

= *uliginosa* (Fallén, 1825) preocc.

= *punctata* (Robineau-Desvoidy, 1830)

*Helina spinicosta* (Zetterstedt, 1845)

*Helina squalens* (Zetterstedt, 1838)

= *borealis* (Zetterstedt, 1838)

*Helina subvittata* (Séguy, 1923)

= *rothi* Ringdahl, 1939

= *marmorata* auct. nec (Zetterstedt, 1860)

*Helina tetrastigma* (Meigen, 1826)

= *flagripes* (Rondani, 1866)

*Helina trivittata* (Zetterstedt, 1860)

= *atripes* (Meade, 1889)

*Helina veterana* (Zetterstedt, 1838)

*Helina vicina* (Czerny, 1900)

= *suecica* Ringdahl, 1924

***LOPHOSCELES*** Ringdahl, 1922

*Lophosceles cinereiventris* (Zetterstedt, 1845)

= *cristata* (Zetterstedt, 1845)

*Lophosceles frenatus* (Holmgren, 1872)

*Lophosceles hians* (Zetterstedt, 1838)

*Lophosceles mutatus* (Fallén, 1825)

***PHAONIA*** Robineau-Desvoidy, 1830

= *Wahlgrenia* Ringdahl, 1929

= *Dialytina* Ringdahl, 1945

*Phaonia aeneiventris* (Zetterstedt, 1845)

= *cinctinervis* (Zetterstedt, 1860)

*Phaonia alpicola* (Zetterstedt, 1845)

*Phaonia amabilis* (Meigen, 1826)

*Phaonia amicula* Villeneuve, 1922 (see Notes)

*Phaonia angelicae* (Scopoli, 1763)

= *basalis* (Zetterstedt, 1838)

*Phaonia angulicornis* (Zetterstedt, 1838)

= *erinacea* (Fallén, 1824)

*Phaonia apicalis* Stein, 1914

*Phaonia atriceps* (Loew, 1858)

*Phaonia atrocyanea* Ringdahl, 1916

*Phaonia canescens* Stein, 1916

*Phaonia consobrina* (Zetterstedt, 1838)

*Phaonia czernyi* Hennig, 1963

= *steinii* Czerny, 1900 preocc.

*Phaonia errans* (Meigen, 1826)

= *erratica* (Fallén, 1825) preocc.

*Phaonia erronea* (Schnabl, 1887)

*Phaonia falleni* (Michelsen, 1977)

= *vagans* (Fallén, 1825) preocc.

*Phaonia fugax* Tiensuu, 1946

*Phaonia fuscata* (Fallén, 1825)

*Phaonia gobertii* (Mik, 1881)

*Phaonia gracilis* Stein, 1916

*Phaonia grandaeva* (Zetterstedt, 1845)

*Phaonia halterata* (Stein, 1893)

*Phaonia hybrida* (Schnabl, 1888)

*Phaonia incana* (Wiedemann, 1817)

*Phaonia jaroschewskii* (Schnabl, 1888)

= *crinipes* Stein, 1913

*Phaonia kowarzii* (Schnabl, 1886)

= *fulvicornis* Tiensuu, 1936

*Phaonia laeta* (Fallén, 1823)

*Phaonia latipalpis* Schnabl, 1911

= *umbraticola* d’Assis-Fonseca, 1957

*Phaonia longicornis* Stein, 1916

*Phaonia lugubris* (Meigen, 1826)

= *morio* (Zetterstedt, 1845)

*Phaonia magnicornis* (Zetterstedt, 1845)

*Phaonia meigeni* Pont, 1986

= *lugubris* auct. nec (Meigen, 1826)

*Phaonia mystica* (Meigen, 1826)

= *vittifera* (Zetterstedt, 1845)

*Phaonia nymphaearum* (Robineau-Desvoidy, 1830)

= *nitida* (Macquart, 1835)

*Phaonia pallida* (Fabricius, 1787)

*Phaonia pallidisquama* (Zetterstedt, 1849)

*Phaonia palpata* (Stein, 1897)

*Phaonia perdita* (Meigen, 1830)

*Phaonia pratensis* (Robineau-Desvoidy, 1830)

= *laeta* auct. nec (Fallén, 1923)

*Phaonia rufipalpis* (Macquart, 1835)

*Phaonia rufiventris* (Scopoli, 1763)

= *populi* (Meigen, 1826)

*Phaonia serva* (Meigen, 1826)

*Phaonia steinii* (Strobl, 1898)

*Phaonia subfuscinervis* (Zetterstedt, 1838)

*Phaonia subventa* (Harris, 1780)

= *variegata* (Meigen, 1826)

*Phaonia taigensis* Zinovjev, 1987

*Phaonia tiefii* Schnabl, 1888

*Phaonia trimaculata* (Bouché)

= *servaeformis* Ringdahl, 1916

*Phaonia tuguriorum* (Scopoli, 1763)

= *signata* (Meigen, 1826)

*Phaonia valida* (Harris, 1780)

= *viarum* (Robineau-Desvoidy, 1830)

= *erratica* auct. nec (Fallén, 1825)

*Phaonia villana* Robineau-Desvoidy, 1830

= *mystica* auct. nec (Meigen, 1826)

*Phaonia vivida* (Rondani, 1870)

= *austriaca* (Czerny, 1900)

*Phaonia wahlbergi* Ringdahl, 1930

*Phaonia zugmayeriae* (Schnabl, 1888)

?= *humeralis* (Zetterstedt, 1845)

= *humerella* (Stein, 1900)

## Excluded species

*Coenosia femoralis* (Robineau-Desvoidy, 1830) misidentified

*Fannia barbata* (Stein, 1892) not found within present borders

*Fannia lineata* (Stein, 1895) not found within present borders

*Fannia nigra* Malloch, 1910 not found within present borders

*Hydrotaea glabricula* (Fallén, 1825)

*Limnophora pollinifrons* Stein, 1916 not found within present borders

*Phaonia bitincta* (Rondani, 1866) misidentified

*Scathophaga calida* Haliday in Curtis, 1832 not found within present borders

*Spilogona acrostichalis* (Stein, 1916)

*Spilogona norvegica* (Ringdahl, 1932)

*Spilogona obscuripennis* (Stein, 1916) not found within present borders

*Spilogona septemnotata* (Zetterstedt, 1845) misidentified

*Spilogona setigera* (Stein, 1907)

*Spilogona spininervis* (Villeneuve, 1922)

## Notes

***Coenosia comita* (Huckett, 1936)**. Collected only from Salla. The type locality of *Coenosia ovulivera* Tiensuu, Vuorijärvi, is now Russian territory (Tiensuu 1938). A second specimen, also taken in 1936 by R. Krogerus (in MZH), bears only the label ’Salla’ and probably also originates from the vicinity of Vuorijärvi, but it is possible that it was collected from the Finnish part of Salla, a municipality split between Russia and Finland in 1944.

***Helina ciliata* Karl, 1929**. Ringdahl (1952), quoting ”Tiensuu’s papers”, lists this species from Finland. We could not locate the original record in Tiensuu’s published works or among the available collection specimens. It seems possible Ringdahl make a mistake in his tables. The species is also listed as Finnish by Hackman (1980), again without any details.

***Limnophora pandellei* Séguy, 1923**. Only females have been found in Finland. The identification remains slightly doubtful (Kahanpää 2013).

***Norellia tipularia* (Fabricius, 1794)**. This species was first recorded from Finland by Winqvist (2011). Šifner (2003, 2008) considers *Norellia tipularia* a nomen dubium and probably synonymous with *Norellia spinipes* Meigen, but Ozerov and Krivosheina (2011) disagree and provide a detailed diagnosis.

***Parallelomma paridis* Hering, 1923**. This name was synonymized with *Parallelomma vittatum* (Meigen) by Šifner (1978). Ozerov (2010, 2014) examined the types of *Parallelomma paridis* and upheld the synonymy. Not everyone agrees with this synonymy (Nelson 1990). Finnish CO1 DNA fingerprints (Kahanpää, unpublished) of larvae on *Paris* (Liliaceae) and adults caught among *Dactylorhiza* (Orchidaceae) suggest that two species are involved.

***Phaonia amicula* Villeneuve, 1922**. New to Finland. Recorded as Phaonia sp. nr. halterata by Winqvist (2011): *Ab*: Turku, Ruissalo (67093:32336), 3.vii.2008, leg K. Winqvist, 1 male.
